# Targeting chemokine signaling networks for therapeutics in skeletal disorders

**DOI:** 10.3389/fendo.2025.1667440

**Published:** 2025-12-03

**Authors:** Wenjie Gao, Zhiheng Gao, Yu Chen, Yu Wang, Yonggang Li, Yuchen Qian, Haowen Lu, Xuwei Ling, Heting Xiao, Peng Yang, Yusen Qiao

**Affiliations:** Department of Orthopaedics, The First Affiliated Hospital of Soochow University, Suzhou, China

**Keywords:** chemokine networks, bone remodeling, osteoimmunology, osteoporosis, osteoarthritis, bone regeneration, targeted therapy

## Abstract

Chemokine signaling networks have emerged as pivotal regulators of skeletal homeostasis, integrating inflammation, angiogenesis, and immune activation with the processes of bone remodeling and regeneration. Recent evidence demonstrates that dysregulated chemokine–receptor interactions, including CCL2/CCR2, CCL5/CCR5, and CX3CL1/CX3CR1, disrupt the equilibrium between osteogenesis and osteoclastogenesis, thereby contributing to the pathogenesis of osteoporosis, osteoarthritis, multiple myeloma, and bone metastasis. This review synthesizes current insights into how chemokine-mediated signaling cascades intersect with canonical pathways such as JAK/STAT3, NF-κB, PI3K/Akt, and Wnt/β-catenin to coordinate cellular communication within the bone microenvironment. Furthermore, it highlights recent progress in therapeutic strategies targeting chemokine axes to mitigate inflammatory bone loss and promote tissue regeneration, while addressing translational barriers including receptor redundancy, context-dependent specificity, and limited *in vivo* validation. By positioning chemokines as dynamic mediators at the interface of the immune and skeletal systems, this review establishes a conceptual foundation for the development of precision therapeutics aimed at restoring bone homeostasis and treating skeletal disorders.

## 1 Chemokine species and chemokine receptors in bone tissue

The main chemokines involved in bone tissue remodeling include chemokine 2 (CCL2), chemokine 4 (CCL4), and chemokine 8 (CCL8), which are secreted by osteoblasts and play key roles in bone development and maintenance of bone structure. These chemokines regulate the recruitment and adhesion of osteoclast precursors and immune cells to the bone surface, thereby promoting cellular communication, proliferation, and differentiation. In normal osteoblasts, chemokines bind to their corresponding receptors to activate downstream signaling pathways that sustain bone formation. However, when osteoblasts are injured, they secrete fewer chemokines, leading to impaired cell recruitment and increased bone tissue damage. Thus, the effects of chemokines on osteoblasts and osteoclasts may influence the progression of bone-related diseases.

Chemokines exert their biological effects by binding to specific chemokine receptors expressed on osteoblasts and osteoclasts, thereby activating downstream signaling pathways. Among them, CCL2 and CCL8 play particularly important roles in regulating bone remodeling. In osteoblasts, CCL2 promotes osteogenic differentiation and angiogenesis, supporting bone formation and vascularization. Conversely, in osteoclasts, CCL8 enhances osteoclast differentiation and resorptive activity, contributing to bone degradation. Although both chemokines are involved in bone metabolism, they exert distinct and sometimes opposing functions in osteoblasts and osteoclasts. Further studies are therefore required to elucidate the precise mechanisms by which CCL2 and CCL8 regulate bone homeostasis and their potential implications in bone-related diseases (1).

**Figure 1:**
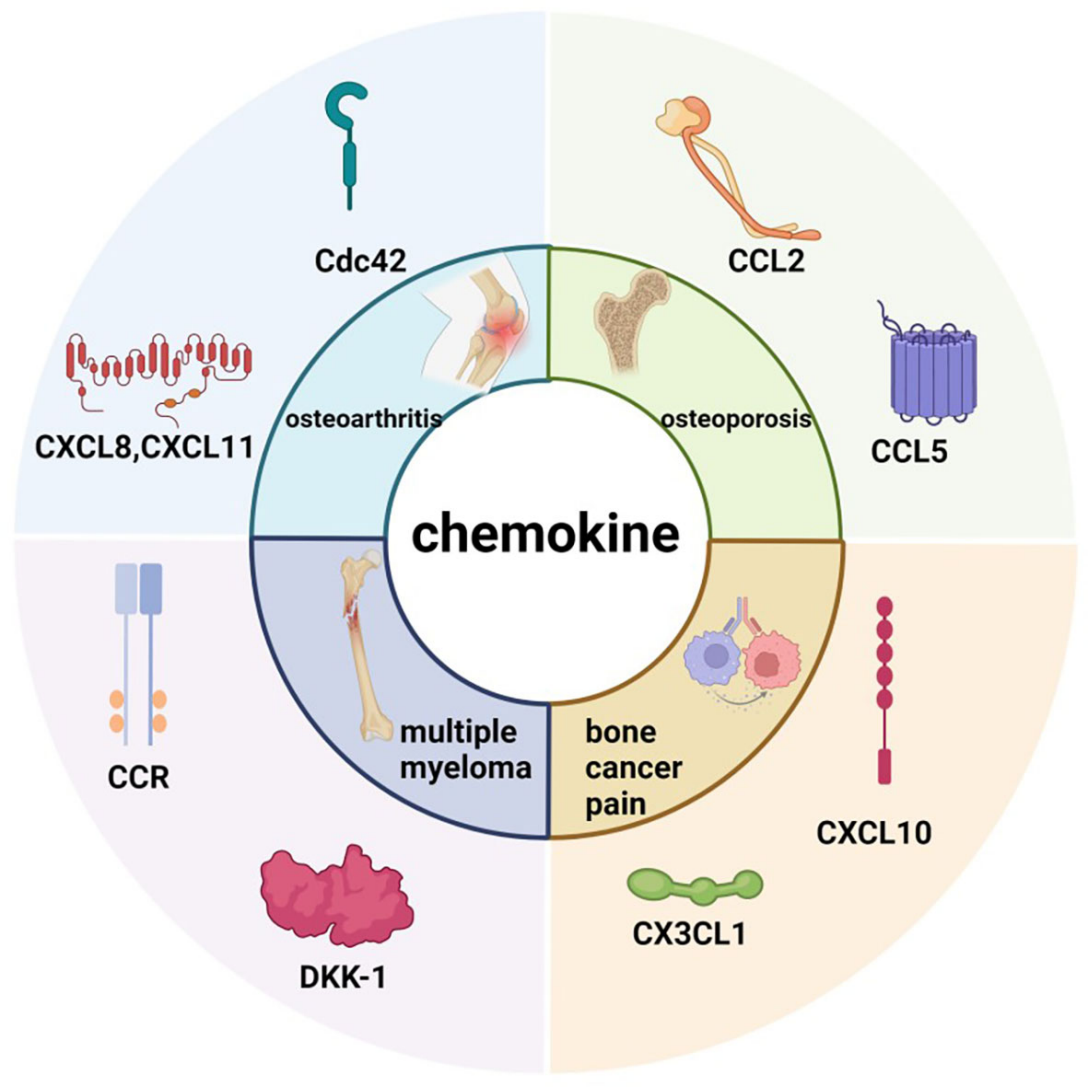
Common chemokines in bone tissue and their abnormal causes of disease.

The interactions between chemokines and their receptors are highly complex and redundant, as several different chemokines can bind to the same receptor, and some chemokines are capable of binding to multiple receptors (2). Many chemokine receptors have been identified. Several chemokine receptors, including CCR1, CCR3, CCR4, and CCR5, are closely associated with tissue and organ lesions in both humans and mice (3). They have been shown to play a role in a variety of diseases, including bone tumors, inflammatory arthritis, chronic inflammation, psoriasis, and Crohn’s disease. These receptors mediate the recruitment and activation of immune cells in response to chemokines such as CCL2, CCL3, CCL5, CCL20, CXCL13, and CX3CL1, thereby contributing to pathological changes and representing potential therapeutic targets. In patients with multiple sclerosis (MS), aberrant activation of specific chemokine–receptor pairs—including CCL2/CCR2 (4), CCL3/CCR1 (5), and CCL5/CCR5 (6)—promotes the infiltration of monocytes, macrophages, and Th1 cells into central nervous system (CNS) lesions, thereby driving acute inflammation and demyelination. The CXCL13/CXCR5 axis is positively correlated with the accumulation of B cells and plasma cells and serves as a predictor of disease activity (7), whereas the CCL20/CCR6 pathway facilitates the migration of pathogenic Th17 cells into the CNS (8). Elevated levels of CX3CL1 and its receptor CX3CR1 further enhance the recruitment of CX3CR1^+^ CD4^+^ T cells, amplifying neuroinflammatory responses (9). Evidence from experimental autoimmune encephalomyelitis (EAE) models supports these mechanisms: CCR2-deficient mice fail to develop typical CNS lesions (4), while pharmacologic blockade of CCL3 or CCR1 markedly reduces T cell and macrophage infiltration (5). Likewise, CCR6 or CCR4/CCR6 double knockout mice display increased resistance to EAE (10), and genetic deletion or inhibition of CXCL13 or CX3CL1 significantly attenuates gliosis and inflammatory damage (11). To visually illustrate the network of key chemokines in bone tissue, **Figure 1** summarizes the distribution of common chemokines during bone remodeling and the mechanisms by which their dysregulation contributes to disease.

## 2 The role of chemokines and their related regulatory molecules in bone diseases

### 2.1 Osteoarthritis

#### 2.1.1 Changes in CXCL8 and CXCL11

**Figure 2:**
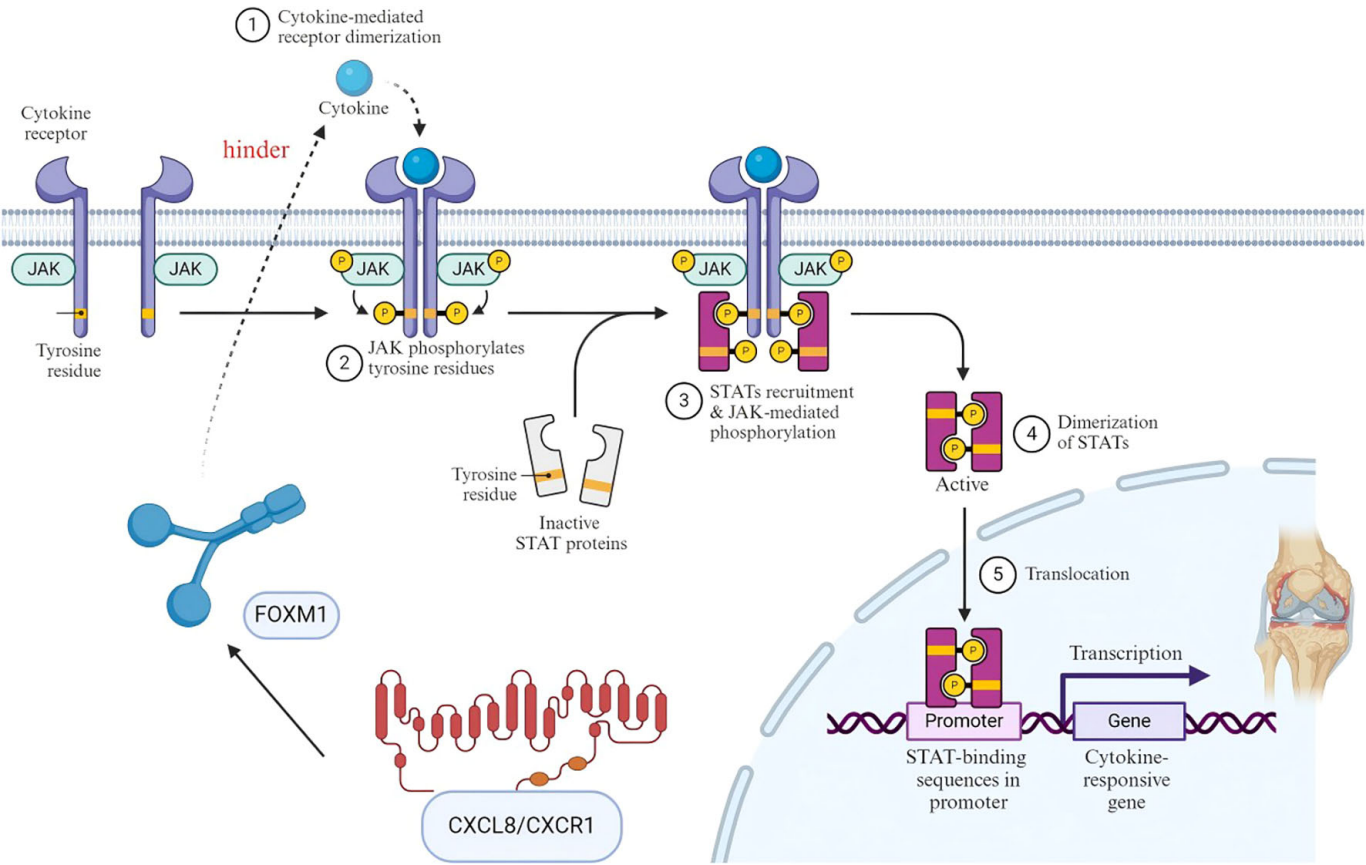
The specific mechanism of CXCL8 and CXCL11 through the JAK/STAT3 signaling pathway.

CXCL8 is the most potent human neutrophil-attracting chemokine and plays a crucial role in the response to infection and tissue injury. CXCL8 activity is intrinsically dependent on interactions with the human CXC chemokine receptors CXCR1 and CXCR2, the atypical chemokine receptor ACKR1, and glycosaminoglycans. In osteoarthritis (OA), CXCL8 and CXCL11 affect chondrocyte apoptosis and proliferation through the JAK-STAT and NF-κB signaling pathways (12). Understanding the fundamental roles of these two signaling pathways and their interactions with CXCL8 and CXCL11 is essential. The JAK/STAT3 pathway is a critical intracellular signaling cascade that regulates diverse cellular processes, including inflammatory responses and cell proliferation (13). In OA studies, activation of the JAK/STAT3 signaling pathway was associated with chondrocyte injury, inflammatory responses, and changes in subchondral bone (14). First, during chondrocyte apoptosis, FOXM1 is involved in the inflammatory response of human OA chondrocytes by activating the JAK1/STAT3 pathway (15), and it is now well established that CXCL8/CXCR1 drives the FOXM1 protein to translocate relevant inflammatory factors (16). Collectively, these findings suggest that CXCL8/CXCR1-mediated activation of the JAK1/STAT3 signaling pathway may involve FOXM1 as an intermediate regulatory factor, a hypothesis that warrants further experimental validation to establish a definitive mechanistic link. For example, CXCL8 can promote chondrocyte proliferation and antiapoptotic signaling by activating the JAK/STAT3 signaling pathway (17), and CXCL11 may regulate chondrocyte function by affecting the NF-κB signaling pathway (18). In clinical treatment, diacerein, an anthraquinone derivative, can reduce chondrocyte apoptosis by activating the JAK/STAT3 signaling pathway (19). In the inflammatory response, the JAK/STAT3 signaling pathway is activated in a variety of cell types, including macrophages and endothelial cells; for example, STAT3 signaling activation in macrophages is correlated with activation of the NLRP3 inflammasome, which is associated with inflammatory bone loss (20). Finally, subchondral bone changes occur, where the activation of Stat3 in subchondral bone H-vessels leads to greater cell proliferation, migration, and angiogenesis, which may exacerbate the progression of OA (21).To provide a detailed view of the molecular mechanisms of CXCL8 and CXCL11 in osteoarthritis, **Figure 2** depicts how these chemokines influence chondrocyte apoptosis and proliferation through the JAK/STAT3 signaling pathway.

The NF-κB signaling pathway plays an important role in the development of osteoarthritis. NF-κB is a transcription factor that, when activated in the nucleus of a cell, can regulate the expression of a variety of genes involved in inflammatory responses, cell proliferation, and apoptosis. The activation of NF-κB leads to the proliferation and differentiation of chondrocytes and participates in the process of cartilage degradation. Although direct evidence is scarce, these chemokines may indirectly influence chondrocyte behavior by affecting the two signaling pathways mentioned above.

*In vitro* studies have provided direct evidence for the involvement of CXCL8 and CXCL11 in activating the NF-κB signaling pathway in OA chondrocytes. When OA patient-derived chondrocytes were treated with CXCL8 (10 ng/mL), a significant increase in nuclear translocation of the NF-κB p50 subunit was observed, accompanied by elevated phosphorylation of IκBα, indicating classical NF-κB pathway activation (12). Similarly, treatment with CXCL11 (100 ng/mL) not only induced p50 nuclear translocation but also enhanced IKK activity via the JNK MAPK pathway, forming a positive feedback loop that sustains NF-κB activation. Furthermore, blocking CXCL11 with a neutralizing antibody reduced p65 phosphorylation by approximately 70% and significantly suppressed the secretion of proinflammatory cytokines such as IL-17A (22) with key findings summarized in **Table 1**.

**Table 1:** Summary of CXCL8/CXCL11 signaling in OA.

Chemokine	Passage	Experimental model and discovery	Reference
CXCL8	JAK/STAT3	CXCL8 stimulated p-STAT3 and promoted the expression of MMP-13 and IL-6 in chondrocytes. Gene silencing inhibits STAT3 activation	(17)
	NF-κB	NF-κB p65 binds to CXCL8 promoter; The inhibitor BAY11–7082 blocked CXCL8 expression	(23, 24)
CXCL11	JAK/STAT3	p-STAT3 increased after chondrocyte stimulation. Neutralizing antibodies reduce M1 macrophage-dependent STAT3 signaling	(25)
	NF-κB	TNF-α directly activates CXCL11 transcription through NF-κB; The CXCR3-PKC/MAPK pathway enhances NF-κB nuclear translocation	(26)

#### 2.1.2 Changes in CCL2 and CCL5 in osteoporosis

In osteoporosis, chemokines that affect osteoblast and osteoclast activity include CCL5, CCL3, and CCL4. These chemokines play important roles in regulating osteoblast and osteoclast activity. Their expression, however, is not static; it is dynamically modulated by a variety of intrinsic and extrinsic factors. Pro-inflammatory cytokines, including TNF-α and IL-1β, can upregulate these chemokines, promoting osteoclastogenesis and altering the local bone microenvironment, while mechanical stimuli have been shown to specifically drive CCL3 expression, linking physical loading to chemokine-mediated bone adaptation. Hormonal regulation, particularly estrogen deficiency, further contributes to chemokine dysregulation, exacerbating the imbalance between bone formation and resorption (27, 28).

In osteoporosis, dysregulation of the CCL2/CCL5 ratio is associated with disease progression and may contribute to the pathophysiology of bone loss. CCL2 and CCL5 are expressed mainly on osteoblasts and regulate osteoclast activity through paracrine effects. Dysregulation of the CCL5/CCL5 ratio is associated with elevated serum levels of CCL2 in osteoporosis patients (29). In addition, chemokines that inhibit bone resorption affect osteoblast activity. In bone tumors, the expression of various cytokines in the bone tumor microenvironment is also regulated. These factors include chemokines produced by stromal cells, osteoconversion-associated chemokines, and chemokines produced by osteoblasts. These factors are upregulated in bone tumors to stimulate cancer cell proliferation and metastasis. Recent studies have shown that the inhibition of chemokines produced by osteoblasts suppresses bone tumor growth and metastasis. In addition, bone microstructural destruction has been associated with a variety of inflammatory and immune molecules in these diseases (30). These factors include interleukin(IL)-6, IL-8, IL-6, and TNF-α. Studies have shown that these inflammatory molecules also play important roles in bone tumor growth.

In osteoblasts, the activation of CCR2 by CCL2 has been shown to trigger multiple signaling pathways. One of the major pathways is the phosphatidylinositol 3 - kinase (PI3K)/Akt pathway. The activated G - protein βγ subunits can directly interact with and activate PI3K. PI3K phosphorylates phosphatidylinositol - 4,5 - bisphosphate (PIP2) to phosphatidylinositol - 3,4,5 - trisphosphate (PIP3), which recruits Akt to the plasma membrane. At the membrane, Akt is phosphorylated and activated by kinases such as phosphoinositide - dependent kinase - 1 (PDK1). Activated Akt then phosphorylates various downstream targets, including glycogen synthase kinase - 3β (GSK - 3β). Inhibition of GSK - 3β by Akt phosphorylation can lead to increased β - catenin stability and nuclear translocation. In the nucleus, β - catenin interacts with T - cell factor/lymphoid enhancer - binding factor (TCF/LEF) transcription factors, regulating the expression of genes involved in osteoblast differentiation and function, such as Runx2 and osterix.

In osteoclasts, CCL5 binding to its receptors, particularly CCR5, is important for osteoclastogenesis and function. CCL5 - CCR5 interaction activates the NF - κB pathway. When CCL5 binds to CCR5, the associated G - protein is activated. The activated G - protein can lead to the activation of inhibitor of κB kinase (IKK) complex. IKK phosphorylates the inhibitor of κB (IκB) protein, causing its degradation. This releases the NF - κB transcription factor, which then translocates to the nucleus. In the nucleus, NF - κB binds to specific DNA sequences in the promoter regions of genes involved in osteoclast differentiation and function, such as nuclear factor of activated T - cells, cytoplasmic 1 (NFATc1). NFATc1 is a master regulator of osteoclastogenesis, and it’s up - regulation by CCL5 - induced NF - κB activation promotes osteoclast differentiation and bone resorption.

### 2.2 Multiple myeloma

#### 2.2.1 Alterations in chemokine receptors

Multiple myeloma (MM) is a hematologic malignancy, and its therapeutic efficacy is related to a variety of factors, including patient age, sex, and disease stage. In recent years, researchers have focused on the expression of the chemokine receptor in MM and its relationship with treatment outcome.

The expression levels of chemokine receptors such as CXCR4, CCR 1, and CCR2 in MM are closely related to the prognosis of the disease. For example, high expression of CXCR4 is associated with poor prognosis in MM patients. CXCR4 affects disease progression by promoting tumor cell migration and settlement, mainly through binding to stromal cell-derived factor-1 (SDF-1) (31). In addition, increased expression of CXCL12/CXCR4 is associated with the onset and progression of MM, and high expression may suggest a poor prognosis for patients.

Conversely, the absence or low expression of chemokine receptors is associated with a better treatment response and longer survival. For example, one study showed that patients with negative chemokine receptor expression had increased progression-free survival and overall survival. These findings suggest that chemokine receptor expression levels may be important predictors of MM treatment outcome (32).

In addition, chemokines and their receptors not only affect the behavior of tumor cells but also indirectly influence therapeutic effects through mechanisms such as the modulation of the immune response and angiogenesis. For example, the expression of CXCR4 is associated with R-ISS staging and the prognosis of MM (33), and the expression of the SOX2 protein and the chemotaxis-related genes CCR1, CCR2, and MCP-1 affect the biological properties of myeloma cells (34). The expression of these genes is significantly related to the expression of chemokine receptors in multiple myeloma and treatment outcomes. High expression of chemokine receptors is usually associated with poorer therapeutic response and shorter survival, whereas low expression may predict better therapeutic outcome and longer survival.

#### 2.2.2 Changes in the levels of DKK-1 and sRANKL

The serum levels of DKK-1 and sRANKL are significantly correlated with the stage of multiple myeloma (MM) and the extent of osteolytic lesions (35).

1. Relationships between DKK-1 levels and MM stage and the extent of osteolytic lesions:

DKK-1 is an inhibitor of the Wnt signaling pathway, and its expression level is significantly higher in multiple myeloma patients than in healthy controls. These findings suggest that DKK-1 may play an important role in the pathogenesis of MM.

According to the International Staging System (ISS), serum DKK-1 levels are significantly higher in patients with stage III MM than in patients with stages I to II (36) MM. In addition, serum DKK-1 levels are significantly higher in patients with osteolytic lesions than in patients without osteolytic lesions (37). These data suggest that DKK-1 levels are closely related to the severity of MM and the extent of osteolytic lesions.

2. Relationships between sRANKL levels and MM stage and the extent of osteolytic lesions:

sRANKL, a key factor that promotes osteoclast activity, is also expressed at significantly higher levels in MM patients than in healthy controls (38). These findings further support the role of sRANKL in the development of MM-related bone disease. Like DKK-1, sRANKL levels are also significantly higher in stage III MM patients than in stage I-II patients (39). In addition, serum sRANKL levels are significantly higher in patients with osteolytic lesions than in patients without osteolytic lesions (40).

3. Relationship between DKK-1 and sRANKL:

There was a positive correlation between the serum levels of DKK-1 and sRANKL. This implies that both may be jointly involved in the pathologic process of MM, especially in the development of bone disease. The serum levels of DKK-1 and sRANKL are not only associated with the stage of multiple myeloma but also closely correlated with the extent of osteolytic lesions. These findings provide a potential clinical biomarker for assessing the severity of MM and the efficacy of treatment.

### 2.3 Changes in CX3CL1 and CXCL10 in bone cancer pain

The mechanism by which fractalkine (CX3CL1) and CXCL10 are involved in bone cancer pain development through the activation of spinal microglia involves multiple steps and signaling pathways (41). First, fractalkine is a unique membrane-bound chemokine that is expressed mainly by neurons and can exist in a soluble form with different biological activities. In the context of bone cancer pain, CX3CL1 interacts with spinal microglia through its receptor CX3CR1 (42), which promotes an inflammatory response and increased pain sensitivity.

Specifically, CX3CL1 is capable of releasing and forming diffusible signals from neurons that activate microglia when it binds to CX3CR1 on these cells. This activation leads microglia to produce a variety of inflammatory mediators, such as IL-1β and TNF-α, which further exacerbate pain perception and transmission (43). In addition, CX3CL1 can participate in the metastatic process of tumors by promoting cell migration and adhesion, as demonstrated in studies of osteosarcoma (44).

## 3 The importance and potential therapeutic strategies of modulating chemokines in bone disease

### 3.1 CX3CL1-CX3CR1 axis - treatment of osteoporosis

CX3CL1 and its receptor CX3CR1 are expressed on osteoblast and osteoclast precursors, respectively. CX3CL1 expression is upregulated upon stimulation with interleukin-1β, while the application of a neutralizing anti-CX3CL1 antibody inhibits both the migration of osteoclast precursors and their subsequent differentiation (45). These findings suggest that CX3CL1 promotes the migration and differentiation of osteoclast precursors by binding to CX3CR1. The CX3CL1-CX3CR1 pathway acts mainly in the early stages of osteoblast differentiation (46). CX3CL1 induces the migration of osteoclast precursor cells within the bone marrow and regulates osteoclast adhesion but does not directly induce osteoclast differentiation. These findings suggest that the CX3CL1–CX3CR1 axis plays an important role in maintaining osteoclast precursor cell aggregation and enhancing bone resorption. CX3CL1 is a transmembrane chemokine with dual functions as an adhesion molecule and a chemotactic agent. The gene encoding CX3CL1 is located on chromosome 16q13, and it is expressed predominantly on activated endothelial cells, activated fibroblasts, and osteoblasts. This dual function enables it to regulate multiple intercellular signaling pathways (47).

Owing to the critical role of the CX3CL1–CX3CR1 axis in the migration and differentiation of osteoclast precursors, as well as its regulatory role during bone resorption and fracture repair, this axis has potential as a therapeutic target. For example, by inhibiting CX3CL1 or its receptor CX3CR1, the formation and activity of osteoclasts can be reduced, thereby slowing bone loss, which is important for the treatment of skeletal diseases such as osteoporosis (48).

**Figure 3:**
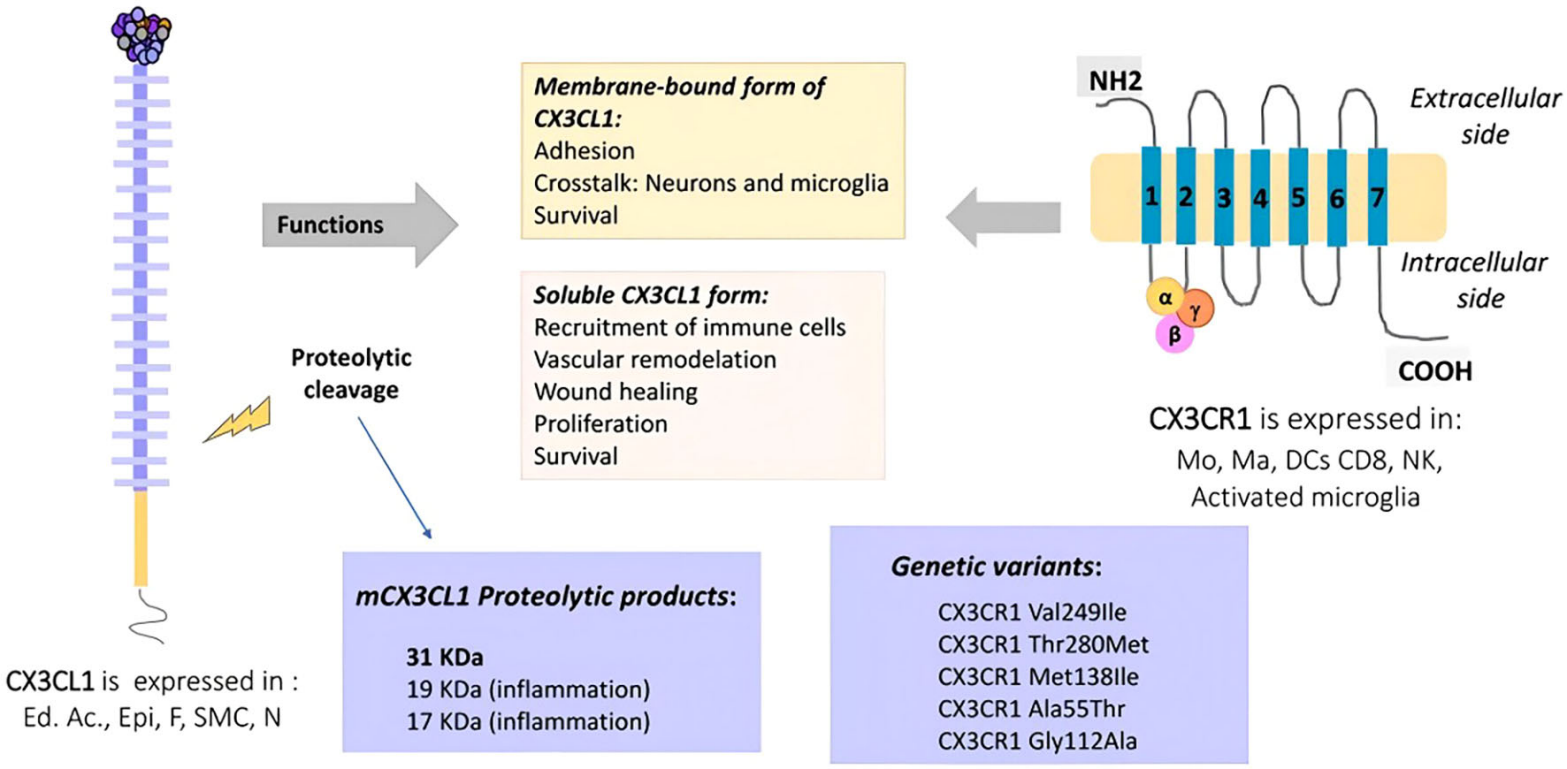
CX3CL1-CX3CR1 normal expression sites and related sites of action.

Recent studies have reinforced the therapeutic potential of targeting the CX3CL1–CX3CR1 axis in bone-related diseases. Administration of anti-CX3CL1 monoclonal antibodies has been shown to suppress the migration and differentiation of osteoclast precursor cells, reduce the number of mature osteoclasts, and markedly alleviate bone loss in ovariectomized (OVX) mouse models of osteoporosis. Furthermore, CX3CR1-deficient mice (Cx3cr1 GFP/GFP) exhibit increased cortical bone thickness and enhanced trabecular bone density in models of inflammatory bone destruction, further supporting the efficacy of receptor-targeted strategies (49). These findings underscore the axis’s role not only in bone remodeling but also in the pathological process of bone loss, highlighting its promise as a viable therapeutic target for diseases such as osteoporosis. The therapeutic potential of the CX3CL1–CX3CR1 axis in osteoporosis is visualized in **Figure 3**.

### 3.2 The SDF-1/CXCR4 axis accelerates bone repair

SDF-1 (stromal cell-derived factor-1) is a low molecular weight chemokine with potent chemotactic effects on bone marrow cells. It is primarily expressed by hypoxia-induced cells and continuously secreted by stromal cells. CXCR4, the sole receptor for SDF-1, belongs to the G protein-coupled receptor (GPCR) family. The specific binding of SDF-1 to CXCR4 facilitates the directional migration of bone marrow-derived mesenchymal stem cells (BMSCs) (50). Hypoxic conditions significantly enhanced the migratory ability of BMSCs, in part by upregulating chemokine receptors CXCR4 and CX3CR1. Exposure to 3% oxygen increased both mRNA and protein expression of CXCR4, which likely contributes to the directed migration of BMSCs toward injured lesions *in vivo* (50, 51). These findings suggest that hypoxia may promote the targeted migration of BMSCs to injury sites through the upregulation of chemokine receptors.

Mechanistically, the SDF-1/CXCR4 axis regulates BMSC migration via the PI3K and Erk signaling pathways. Hypoxia induces phosphorylation of PI3K and Erk1/2, thereby activating downstream signaling, while Jak2 phosphorylation is concurrently suppressed. Inhibition of either PI3K or Erk1/2 individually decreases BMSC migration, whereas concurrent inhibition almost completely suppresses the migratory response, even under SDF-1 stimulation (52). Moreover, pretreatment with SDF-1 activates prosurvival signaling pathways involving Akt and Erk, increases the Bcl-2/Bax ratio, and enhances the secretion of angiogenic factors such as basic fibroblast growth factor (b-FGF) and vascular endothelial growth factor (VEGF). These effects are partially reversed by AMD3100, a CXCR4 antagonist (53).

**Figure 4:**
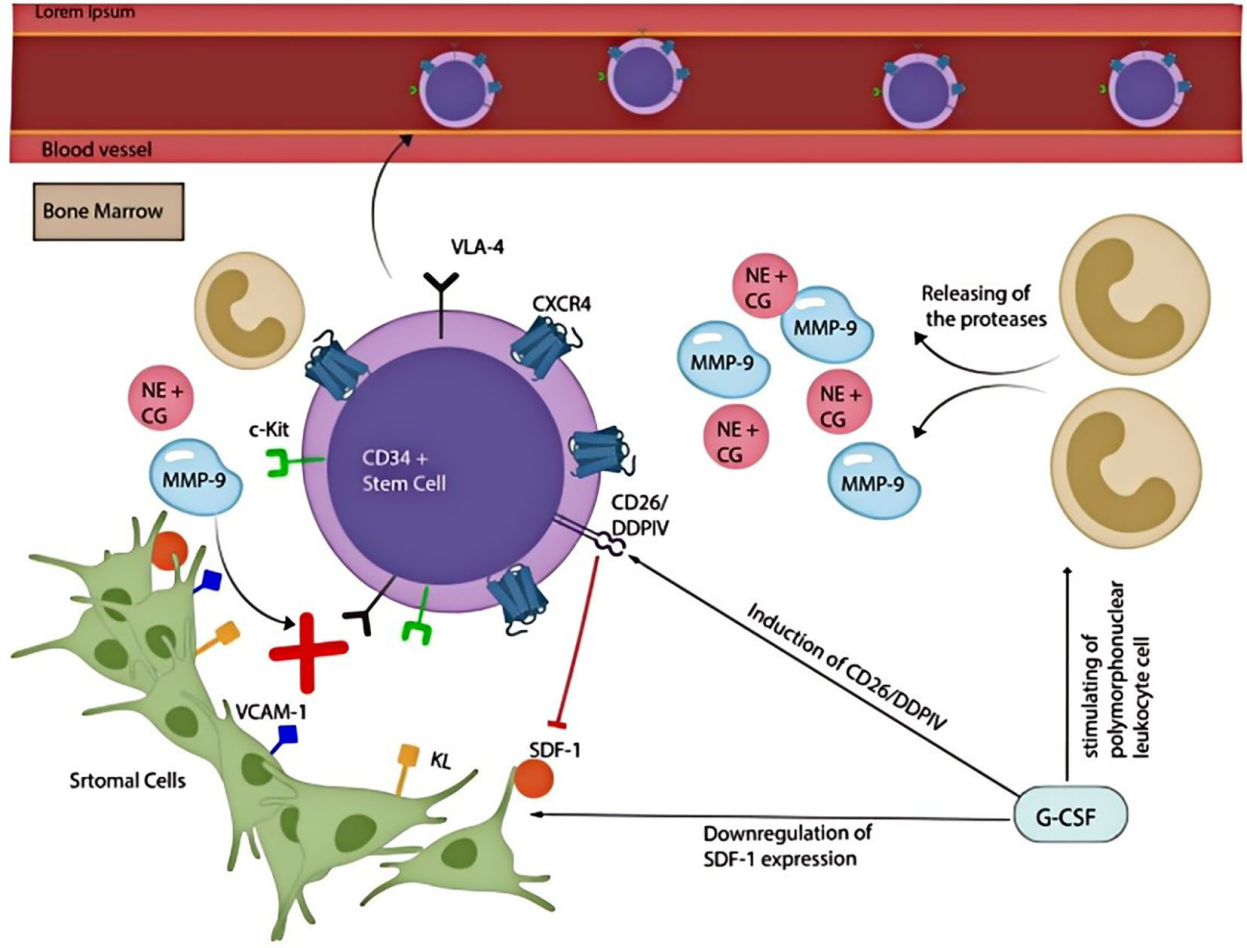
The SDF-1/CXCR4 axis and the mechanisms associated with bone repair.

Recent studies have further emphasized the therapeutic relevance of the SDF-1/CXCR4 axis in bone regeneration, particularly under trauma-associated conditions. In a murine model of traumatic brain injury (TBI) combined with bone fracture, accelerated endochondral bone repair was observed (54). TBI markedly upregulated SDF-1 and CXCR4 expression at the fracture site during the early post-injury phase, facilitating the targeted recruitment of BMSCs to the injured region (55). Both *in vitro* and *in vivo* studies demonstrated that SDF-1 promoted BMSC chemotaxis in a dose-dependent manner, an effect significantly inhibited by AMD3100, confirming the role of CXCR4 signaling. Functional blockade using anti-SDF-1 antibodies or CXCR4 antagonists led to a marked reduction in new bone formation and impaired fracture healing. Collectively, these findings highlight the essential role of the SDF-1/CXCR4 axis in orchestrating MSC recruitment and promoting bone regeneration, suggesting that modulation of this pathway may represent a promising therapeutic strategy for complex injuries involving impaired bone healing (56). The central role of the SDF-1/CXCR4 axis in bone repair is systematically presented in **Figure 4**.

### 3.3 Wnt/β-catenin signaling pathway - bone metabolic diseases

The Wnt/β-catenin pathway serves as an intersecting osteogenic pathway that integrates extracellular cues, intracellular transduction, and therapeutic interventions to maintain bone homeostasis. It regulates osteogenic differentiation of mesenchymal stem cells (MSCs) as well as the proliferation and activity of osteoblasts and osteoclasts. Pathway activation occurs when extracellular Wnt ligands bind the Frizzled and LRP5/6 receptor complex, stabilizing β-catenin and promoting its nuclear translocation. Within the nucleus, β-catenin associates with TCF/LEF transcription factors to induce osteogenic gene expression, including Runx2 and Alpl, while suppressing adipogenic gene programs (57).

In the absence of Wnt ligands, β-catenin is phosphorylated by a destruction complex comprising Axin, APC, GSK-3β, and CK1α, targeting it for proteasomal degradation and preventing transcription of target genes. Wnt engagement activates Dishevelled (Dvl), which disrupts the destruction complex, allowing β-catenin stabilization and nuclear accumulation. The pathway also engages β-catenin-independent signaling via effectors such as c-JUN and AP-1 through JNK, further regulating proliferation, differentiation, and inflammatory responses (58).

In bone metabolism, Wnt/β-catenin enhances osteogenesis by upregulating BMP2 and osteoprotegerin (OPG) while suppressing RANKL-mediated osteoclast activity, thereby promoting bone formation and inhibiting resorption. Physiological stimuli, including mechanical loading, aerobic exercise, and vibration, can further potentiate pathway activity, whereas pharmacological agents such as parathyroid hormone (PTH) and R-spondin2 modulate bone metabolism through Wnt/β-catenin signaling (59).

**Figure 5:**
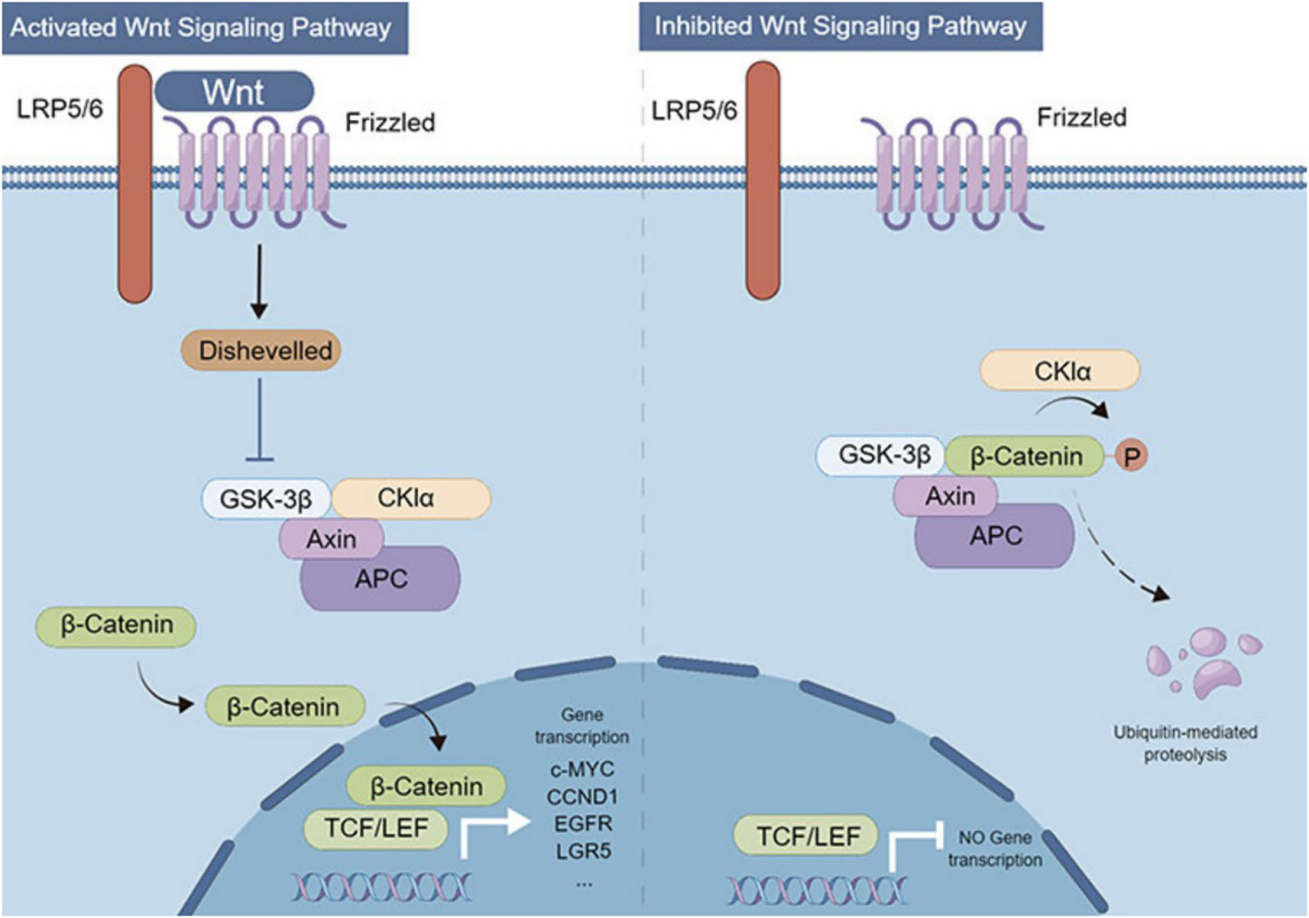
Effects of Wnt signaling-mediated transcriptional regulation on cell carcinogenesis.

Mechanistically, the Mapk7 (ERK5)–Lrp6–β-catenin axis is a critical regulator of MSC fate. Mapk7 phosphorylates Ser1490 on Lrp6, facilitating β-catenin nuclear translocation, upregulating osteogenic gene expression, and suppressing adipogenesis. Inhibition of GSK-3β with CHIR-99021 can rescue osteogenic defects resulting from Mapk7 deletion. Consistently, Mapk7-cKO mice exhibit reduced bone density and increased marrow adiposity, modeling osteoporosis and underscoring the therapeutic potential of targeting this signaling axis (60) as visualized in **Figure 5**.

## 4 Challenges and limitations

In osteoporosis and other metabolic bone disorders, dysregulation of chemokine networks—particularly imbalances such as the CCL2/CCL5 ratio—has emerged as a critical factor driving disease progression and bone loss. Elevated circulating levels of CCL2, CCL3, CCL4, and CCL5 have been reported in patients with osteoporosis, suggesting that excessive chemokine signaling may enhance osteoclast recruitment and inflammatory bone resorption. However, most mechanistic insights have been derived from *in vitro* studies employing chondrocyte or MSC cultures. Although these models are valuable for elucidating specific molecular pathways, they cannot fully recapitulate the complex mechanical, biochemical, and multicellular interactions within the bone microenvironment (29).

Animal studies have provided important evidence linking chemokine signaling to bone remodeling. Nevertheless, substantial translational gaps persist between preclinical models and human pathophysiology. Species-specific variations in immune regulation, bone turnover dynamics, and chemokine receptor expression reduce the predictive relevance of animal data for clinical outcomes, emphasizing the need for well-designed, prospective human studies to validate mechanistic hypotheses (61).

Furthermore, chemokine redundancy and receptor cross-reactivity complicate the development of targeted therapies. Because multiple chemokines can activate the same receptor, and individual receptors often respond to several ligands, these overlapping and compensatory pathways diminish the effectiveness of single-target interventions. This functional redundancy underscores the importance of precision medicine strategies that consider individual variability in chemokine expression and receptor activity (62).

## 5 Outlook

Although the role of chemokines in bone tissue is still an open area of research, studies have shown that they have some therapeutic potential. Chemokines are a class of attractive mediators that help tissues achieve self-renewal and assist the body in the uptake of nutrients such as calcium. Therefore, understanding the mechanism of action of chemokines in these diseases and their potential as therapeutic targets is important. We hope that in the future, we will be able to establish more effective methods to study the relationship between these chemokines and these diseases and to find ways to inhibit the role of these chemokines in these diseases, thus providing new ideas for the development of drugs that effectively treat these diseases. To systematically summarize emerging therapeutic targets, **Table 2** provides an overview of other promising chemokines currently under investigation for bone disease modulation.

**Table 2:** Other promising chemokines.

Chemokines	Action process	Function characteristics	Reference
CCL4	Bone Remodeling	• Inhibition of osteoclast migration induced by RANKL, • Stimulate osteoclast production	(2)
CXCL12/CXCR4	Maintenance CSC	Induction of CSC in PCa cells	(63)
CXCR1/CXCR2	Tumor heterogeneity, stem cell maintenance and metastasis	Self-renewing pancreatic CSC	(64)
ChemR23	Temporomandibular joint osteoarthritis	May play a key role in joint inflammation and cartilage destruction	(65)
CCL19 and CCL21	Inflammatory bone destruction	Promote osteoclast migration and absorption activity	(66)
CCL21	The development of myeloma	Resulting in poor T cell activation and adverse effects on the immune system	(67, 68)
RANTES (CCL5)	Bone marrow defect of jaw	May be a key regulator in promoting DDw/OR-induced osteoclast generation	(69, 70)
CXCL8	Bone metastasis in prostate cancer	Bone metastases are present in only a few cases, and higher CXCL8 levels may be associated with an increase in T cell inhibitory myeloid suppressor cells (MDSC), a phenomenon that may contribute to bone metastases	(71)
